# Features and impact of missing values in the association of self-rated health with mortality in care homes: a longitudinal study

**DOI:** 10.1186/s12955-019-1184-z

**Published:** 2019-06-29

**Authors:** María del Pilar Rodríguez-García, Alba Ayala, Carmen Rodríguez-Blázquez, Pablo Martínez-Martín, Maria João Forjaz, Javier Damián

**Affiliations:** 1 Puertollano Integrated Care Management, Ciudad Real, Spain; 2 National School of Public Health, Institute of Health Carlos III and REDISSEC, Avda Monforte de Lemos 5, 28029 Madrid, Spain; 3 National Center of Epidemiology, Institute of Health Carlos III and CIBERNED, Madrid, Spain

**Keywords:** Self-rated health, Mortality, Care and nursing homes, Elderly people, Missing values

## Abstract

**Background:**

Self-rated health (SRH) is a health measure used in studies of older adults. The objective of this study is to analyze SRH as a predictor of mortality in the institutionalized older population and the characteristics of those who do not provide information about their SRH on health questionnaires.

**Methods:**

This is a 15-year follow-up study of older adult residents in nursing or care homes in of Madrid, Spain. SRH was measured on a 5-point Likert type scale. The association between answering the SRH question and socio-demographic and health characteristics was evaluated through prevalence ratio (PR), estimated by Poisson regression models. Survival rates associated with SRH were studied through a multivariate Cox regression.

**Results:**

The sample has a mean age of 83.4 (standard deviation, SD = 7.3), with 75.7% women. Twelve percent did not answer the SRH item. Those who did not answer showed a higher probability of disability (Barthel index, PR = 0.76, 95% confidence interval = 0.67–0.86) and/or dementia (PR = 8.03, 3.38–19.03). A trend for higher mortality was observed in those persons who did not respond (adjusted hazard ratio HR = 1.26, 0.75–2.11). The mortality rate was 32% higher for those who declared poor SRH in comparison with those who reported good SRH (adjusted HR = 1.32, 1.08–1.6).

**Conclusions:**

There is an elevated number of people who do not respond to the SRH item, mainly those with disabilities and cognitive deterioration. Lack of response to SRH is a good indicator of 15-year mortality for persons institutionalized in care or nursing homes.

## Background

Self-rated health (SRH) is the rating that individuals give to their health status. It is a global measure both of mental state and of physical condition [1]. In 1982, Mossey and Shapiro considered SRH as a predictor of mortality among the older population [2]. Since then, it has frequently been used as an indicator of health and as a predictor of mortality or survival in both the general and older population [3], as it is an easy, low-cost and convenient measure and has been widely validated [4]. It has been recommended as an indicator by the World Health Organization since 1996 for its ability to evaluate mortality, morbidity, functional state, and disability and, consequently, enable effective management of health resources [5].

The ability of SRH to predict the survival of individuals depends on the insight that they have of their own state of health [1]. This situation can explain the variations in SRH as a mortality predictor across distinct population groups, such as by social and cultural class, and by age [1]. As an example, older people have a higher probability of suffering potentially fatal events, which are not taken into account in the perception of their health status. This is because the baseline health status for these people (prior to the adverse event) is lower, which contributes to a weaker association between SRH and mortality at more advanced ages [1].

On the other hand, there are various studies that suggest that the relation between SRH and mortality is significantly weaker when models adjust for other health indicators [3, 6]. Disability or chronic health conditions can be determinant factors for individuals in rating their own health status. Similarly, symptoms of depression or cognitive problems can play an important role [1, 7], both in the general population and in institutionalized people in care or nursing homes [8].

Other studies have explored SRH as a factor associated with survival in specific populations, as is the case with people suffering from dementia [9]. In these cases, people with greater cognitive deterioration may be incapable of evaluating their own health status, which entails a limitation for the analysis [10].

The majority of longitudinal studies on SRH has a follow-up period of between 6 and 9 years [3], and some cover even shorter periods. It is worthwhile studying whether SRH maintains an effective role as a measure of health and predictor of survival over longer follow-up periods. Furthermore, most of the studies that relate SRH and mortality usually use surveys on non-institutionalized population [11, 12], which has a better health status than institutionalized older adults. Our study fills this research gap by focusing on the institutionalized population.

As a consequence, in this study our objective is to study SRH as a predictor of survival in a cohort of institutionalized older people in Madrid for a 15 year follow-up period. In addition, as a second objective, we analyse the frequency of “no response” and the characteristics of the individuals who do not respond to the SRH questionnaire and study their mortality.

## Methods

### Design and participants

A retrospective cohort study was carried out on a sample of 699 people over 65 years old, who lived in care and nursing homes in Madrid, with a 15-year maximum follow-up.

We selected a baseline probabilistic sample of residents, aged 65 years and over, of public and private nursing homes in the city of Madrid (Spain) and a surrounding area of up to 35 km distant. Study participants were selected through stratified cluster sampling, including one stratum with 22 public and 25 subsidized (privately owned but publicly funded) nursing homes and another stratum with 139 private institutions. As a first stage, we sampled 25 public/subsidized and 30 private institutions with probability proportional to their sizes. As a second stage the interviewers obtained a list of all the residents from the director of each facility and then they selected 10 men and 10 women in each public/subsidized facility chosen and five men and five women from each private nursing home chosen by means of a systematic sampling with random start (with the aid of random number tables). Four private institutions (totalling 40 sample subjects) refused participation and 45 additional residents could not be selected due to absence or refusal, leading to an overall response rate of 89% (715 of the 800 sample residents). Due to refusal, prolonged absence or sampling frame errors, 39 subjects were randomly replaced with residents of the same facility and sex, with the consequence that information could be gathered through structured interviews with 754 residents. Of the 754 participants in the baseline survey, 55 with unknown vital status on termination of follow-up were excluded leaving a study sample of 699 people.

### Assessments

All-cause mortality was considered as a main endpoint. Mortality was ascertained by reference to the Spanish National Death Index provided by the Ministry of Health and, in addition, information regarding deaths was obtained from the survey completed by the facilities in 2013 [13]. The baseline data were collected via structured questionnaires between June 1998 and June 1999. These questionnaires were administered by trained interviewers to the residents, medical personnel from the facility, and the principal carer. Socioeconomic information were also collected by the interviewers.

SRH was assessed in the interview administered to the residents via the question “in general terms, how would you rate your health?” The response was collected through a scale with five response options: very good, good, moderate, bad or very bad. Afterwards these were grouped into two categories: good SRH (“very good” and “good”) and bad SRH (“moderate”, “bad” and “very bad”).

The presence of 20 chronic health conditions associated with higher mortality, including chronic pulmonary obstructive disease (COPD), heart failure, diabetes and cancer as well as diagnosis of depression were evaluated through interviews with the medical staff in the facility.

Functional capacity was explored through the Barthel index, as modified by Shah et al. [14], with a rating from 0 to 100. Those residents with scores from 61 to 99 were classified as having a mild or moderate dependence, and 0–60 as severe or total dependence [14]. Information on the Barthel index activities was collected by interviewing the residents’ main caregiver (49%) or the residents themselves when they did not have a caregiver assigned (51%). Data about the cognitive state of the residents were collected indirectly with a question on the dementia, which includes diagnosis of Alzheimer-type dementia and/or other dementias identified in the interview with physician. The presence of pressure ulcers was informed by the physicians [15].

### Data analysis

A descriptive analysis of the data was carried out to study the distribution of socio-demographic variables in the sample (age, sex, marital status, level of education), the presence of chronic problems (COPD, heart failure, diabetes and cancer), and other health indicators (depression, disability, cognitive state and SRH).

We investigated the distribution of chronic problems and health indicators in the residents, grouped by whether or not they answered the SRH question. Subsequently, a multivariate Poisson regression was carried out to measure the association, expressed in prevalence ratios (PR), between the sociodemographic and health variables, and the variable known/unknown SRH. The variables included in the model were age; sex; the presence of pressure ulcers (at the moment of interview); the Barthel index; educational level; and chronic health problems (depression, dementia, COPD, diabetes, cancer, heart failure and others). Finally, survival was analysed for this variable (known/unknown SRH) with a multivariate Cox regression model – in this case adjusted for the same variables present in the Poisson regression model.

Furthermore, 15-year survival was examined in relation to having good or bad SRH. For this, Cox regression model was employed, in this case adjusting for socio-demographic and health variables. We used age instead of follow-up as time scale. A person’s follow-up time depends on the age at which he or she entered the study. Therefore, it was not necessary to adjust the model for this variable. In addition, there are authors who recommend the use of age as a time scale in studies of older people [16, 17]. This model presented 22% of missing data, fundamentally as a result of the SRH variable, but also from other variables. To reduce the probability of selection bias related to this relatively high proportion of missing data, a multiple imputation by chained equations was carried out. This substituted the values of the missing data with a set of simulated values [18]. The model complied with assumption of proportional hazards.

At every stage we used sample weights to re-establish proportionality, and the analysis was carried out considering the complex sampling design. STATA 14 was used to carry out the analysis.

## Results

The 699 individuals who made up the sample had a mean age of 83.4 (standard deviation, SD = 7.3) years at the start of the study (Table 1), 24.3% were men, 14.0% had partners and 14.7% had finished their secondary education or had higher level studies. Almost half lived in public institutions (47%). Over the 15-year follow-up period 598 participants died, which was 84.7% of the total.

**Table 1:** Descriptive analysis

Sociodemographic characteristics			Health problems			Health markers		
	n^a^	%^b^		n^a^	%^b^		n^a^	%^b^
Total	699	100%	COPD			Depression		
Sex			No	520	80.0%	No	546	77.2%
Male	313	24.3%	Yes	172	19.1%	Yes	131	19.4%
Female	386	75.7%	Unknown	7	1.0%	Unknown	22	3.4%
Educational level			CHF			Disability		
Primary or less	551	75.7%	No	548	78.2%	Independent (100)	187	22.1%
Secondary and higher	95	14.7%	Yes	137	19.7%	Mild/moderate (61–99)	316	47.0%
Unknown	53	9.6%	Unknown	14	2.1%	Severe/total (0–60)	179	28.5%
Civil status			Diabetes			Unknown	17	2.5%
With couple	122	14.0%	No	561	81.3%	Dementia		
Without couple	553	81.9%	Yes	135	18.3%	No	494	67.8%
Unknown	24	4.1%	Unknown	3	0.4%	Yes	198	31.2%
Type of residence			Cancer			Unknown	7	1.0%
Public	401	47.0%	No	629	90.8%	Self-rated Health		
Subsidized	72	8.0%	Yes	66	8.7%	Good	336	48.1%
Private	226	45.1%	Unknown	4	0.6%	Bad	292	39.9%
Dead at 15 years			Pressure ulcer			Unknown	71	12.0%
No	81	12.1%	No	677	97.0%		Mean^b^	SD^b^
Yes	598	84.7%	Yes	22	3.0%	Age	83.4	7.3
Unknown	20	3.2%	Unknown	0	0%	CHP	3.2	2.1

^a^ Observed frequencies; ^b^ Weighted estimators; *SD* Standard deviation, *CHP* Chronic health problems, *COPD* Chronic obstructive pulmonary disease, *CHF* Congestive heart failure

According to the baseline data, the participants had a mean (SD) of 3.2 (2.1) chronic health problems. Chronic pulmonary disease had a prevalence of 19.1% and congestive heart failure 19.7%. Almost one fifth (19.4%) of the sample had been diagnosed with depression, and 31.2% had dementia. Pressure ulcers were recorded for 3.0% whereas 22.1% of the participants were totally independent in the activities of daily living. Of the participants, 48.1% declared their perception of health to be good; 12% of participants did not answer.

The results of the multivariate study regarding the characteristics of participants who did not answer the SRH question, expressed as PR, are shown in Table 2. A relationship with disability and dementia can be seen, with a PR of 0.76 and 8.03. That is to say, for every increase of 10 points on the Barthel index, the probability of unknown SRH reduces by 24%.

**Table 2:** Factors associated with unknown self-rated health (multivariate Poisson regression)

Variable		PR	CI (95%)	
Sex (ref. female)		0.25	(0.10–0.63)	
Age (ref. 65–74 years)	75–84	0.13	(0.04–0.45)	
	≥85	0.11	(0.005–0.28)	
Educational level (ref. primary or less)	Secondary and higher	0.68	(0.22–2.13)	
Pressure ulcer (ref. No)		1.15	(0.64–2.07)	
Disability (Barthel index)		0.76	(0.67–0.86)	
CHP (ref. < 2)	≥2	0.94	(0.75–1.33)	
Dementia (ref. No)		8.03	(3.38–19.03)	
Depression (ref. No)		0.59	(0.23–1.47)	
COPD (ref. No)		0.90	(0.51–1.58)	
Cancer (ref. No)		0.97	(0.48–1.95)	
Diabetes (ref. No)		0.68	(0.33–1.41)	
CHF (ref. No)		0.45	(0.18–1.14)	

*PR* Prevalence ratio, *CI* Confidence interval, *ref*. Reference category, *COPD* Chronic obstructive pulmonary disease, *CHF* Congestive heart failure, *CHP* Chronic health problems (excluding COPD, CHF, cancer, diabetes, dementia and depression)

**Fig. 1:**
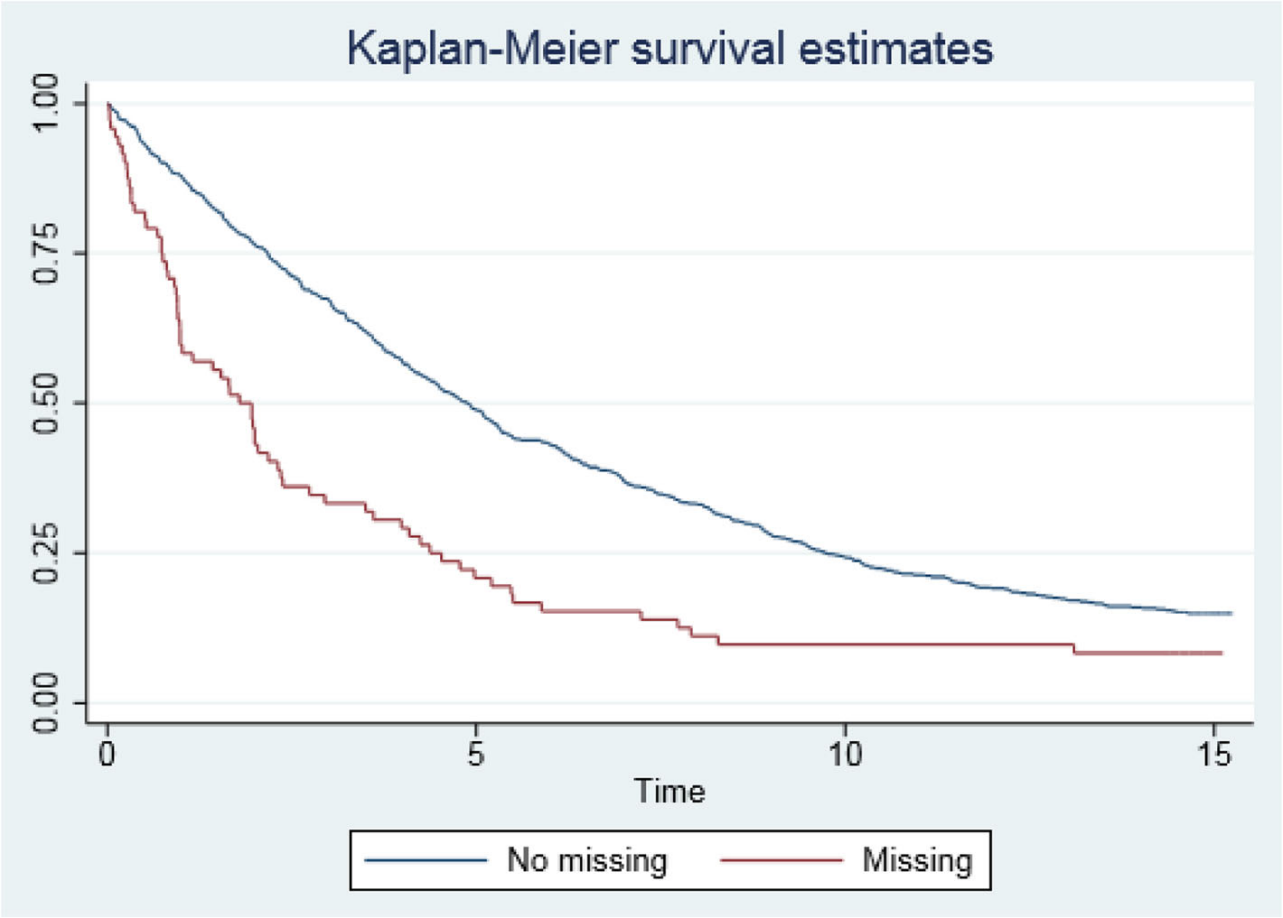
Kaplan-Meier curve for crude mortality relative to reply or not the item self-rated health questionnaire

Figure 1 shows the Kaplan-Meier curve for crude mortality in relation to SRH known and unknown (Hazard Ratio, HR = 1.77; confidence interval, 95%CI = 1.18–2.67). The adjusted value obtained in the Cox regression was HR = 1.26 (95%CI = 0.75–2.11). In other words, those who did not respond to the SRH had a slightly higher risk of dying, although it was not statistically significant at the 5% level.

Finally, Table 3 shows the results of the multivariate Cox regression in relation to SRH (good vs bad). Poor SRH increased the risk of death by 32%, adjusted for sociodemographic variables and for health variables. Using the imputed data, the risk of death for people with poor SRH decreased to 26%.

**Table 3:** Survival based on self-rated health according to the final multivariate Cox regression model including health markers

		Observed model (*N* = 534)			Imputed model (*N* = 699)		
Variable		HR	CI (95%)	*p*-value	HR	CI (95%)	*p*-value
Self-rated health (ref. Good)	Bad	1.32	(1.08–1.60)	0.007	1.26	(1.04–1.53)	0.019
Sex (ref. Female)	Male	1.07	(0.85–1.35)	0.539	1.02	(0.84–1.24)	0.842
Educational level (ref. Primary or less)	Secondary and higher	0.89	(0.63–1.26)	0.491	0.91	(0.66–1.28)	0.588
Civil status (ref. With couple)	Without couple	1.16	(0.84–1.61)	0.361	1.08	(0.81–1.46)	0.584
Type of residence (ref. Public)	Subsidized	0.87	(0.71–1.07)	0.187	0.82	(0.63–1.07)	0.132
	Private	0.72	(0.54–0.96)	0.028	0.73	(0.55–0.97)	0.032
Pressure ulcer (ref. No)	Yes	2.44	(1.19–5.02)	0.017	2.81	(1.61–4.88)	0.001
Disability (ref. Independent)	Mild/moderate	1.25	(0.98–1.58)	0.067	1.31	(1.06–1.62)	0.013
	Severe/total	1.99	(1.39–2.84)	< 0.001	1.98	(1.52–2.58)	< 0.001
Depression (ref. No)	Yes	0.65	(0.49–0.87)	0.004	0.66	(0.51–0.85)	0.002
Dementia (ref. No)	Yes	0.85	(0.61–1.18)	0.312	1.12	(0.84–1.49)	0.421
COPD (ref. No)	Yes	1.30	(0.98–1.74)	0.071	1.36	(1.07–1.73)	0.015
Cancer (ref. No)	Yes	1.20	(0.80–1.79)	0.364	1.23	(0.92–1.64)	0.158
CHF (ref. No)	Yes	1.38	(1.08–1.78)	0.012	1.38	(1.08–1.77)	0.012
Diabetes (ref. No)	Yes	1.39	(1.00–1.92)	0.048	1.22	(0.94–1.59)	0.129
CHP (ref. < 2)	≥2	0.79	(0.58–1.07)	0.123	0.82	(0.65–1.04)	0.104

*CHP* Chronic health problems, *COPD* Chronic obstructive pulmonary disease, *CHF* Congestive heart failure, *CI* Confidence Interval, *HR* Hazard ratio,*ref* Reference category

## Discussion

The construct of SRH has been considered a good predictor of survival in several population groups, like in the over-18 Estonian population [19], or in North Americans over 70 [20, 21]. In this study we have investigated the predictive role of SRH in a cohort of institutionalized people in Madrid over a follow-up period of 15 years. People with poor SRH had a greater risk of death than those who reported good SRH.

### Characteristics of those who did not respond to the questionnaire

In our study we noted that 12% of participants did not answer the SRH question. Other studies have reported values of 18% [20, 21] and 69% [4]. These studies, most of them performed on non-institutionalized population, considered that people who did not answer the SRH question had worse health but did not describe their characteristics.

We found a higher mortality in people who did not report their SRH when compared to those who did answer the SRH question in the questionnaire. Not responding to SRH was related to disability (the greater the degree of disability the higher the prevalence of non-respondents) and to the presence of dementia. This indicates that the non-respondents to the SRH question do have poorer health, which corresponds with previous studies [22]. The non-adjusted mortality risk for non-respondents to this item was higher than for those who did respond. However, in the adjusted model this association was weaker. This suggests that the mortality risk related to answering or not responding to the question is due to, for the most part, disabilities and the presence of cognitive deterioration.

The limitation entailed by cognitive deterioration and the presence of dementia for answering self-report questions in surveys has already been confirmed [23]. There is an association between the level of cognitive deterioration and the proportion of non-responders to self-administered questionnaires (missing data). When there is a high level of missing data, this is considered as study limitation [24] because it hinders the results interpretation.

On the other hand, in the case of our study, people with disability, independently of their cognitive ability, had higher levels of non-response to the SRH question. Nevertheless, the relationship between disability and non-response to self-administered questionnaires is less studied. Generally, studies based on questionnaires usually have disability, especially cognitive, as an exclusion criterion, or a requirement that any disability should be minimal [25, 26]. This means that the selected samples are biased [27]. Therefore, authors like Paula Diehr recommend including all sample members as far as possible [28].

### Other variables related to higher mortality

In the final regression model of this study, the relationship of SRH to mortality was adjusted by other variables related to chronic processes, sociodemographic variables, and health indicators. Some of these variables are also associated with lower survival rates. The characteristics that were related to higher mortality were residing in a public facility rather than in a private one, the presence of pressure ulcers, disability, and heart failure.

Pressure ulcers are related to a severe deterioration in health state and with a lack of care, and thus to higher mortality [29]. Nonetheless, some studies associate this to dementia, disability or comorbidity [30]. In our case, the study has adjusted for all of these variables and the relation of pressure ulcers to mortality is independent of these. This indicates the importance that the care provided has, in order to avoid the occurrence of pressure ulcers – given that they are preventable in 98% of cases [31] – and to improve survival for these people. Indeed, the appearance of pressure ulcers is an indicator of the quality of care, as they are the most preventable and treatable complication presented by people with reduced mobility [31].

The relation of disability, depression and heart failure with mortality has been put forward in numerous studies [32–34]. In our study, these relationships were significant in both models with the observed and imputed data, respectively, which gives greater strength to our results. The finding of higher mortality for people not diagnosed with depression may be because these people are identified, diagnosed and treated unlike other people who may have undetected and untreated depression and, therefore, lower survival [35]. For another two variables, COPD and diabetes, the statistical relationship showed minimal variation between the two models (observed and imputed data). These health conditions are also associated with higher mortality in the literature, as third and fourth highest causes of death in the world [36].

Finally, in our study we found an association between higher mortality and living in a public nursing or care home. The characteristics of the home are important for the residents and the contextual factors that influence this relationship was analysed in another study about facility ownership and mortality [37].

### Strengths and limitations

This study has a number of limitations, the most important of which is the quantity of missing data (22%) at the moment of interpreting the statistical analysis. Nevertheless, the comparison between the imputed and observed data suggests that the observed data was not unduly influenced by the characteristics of the missing data. The second limitation is that the sample is only representative of older adults institutionalized in Madrid, for which reason it would be interesting to repeat the study in other institutionalized older populations. Finally, some deaths could not be identified by the study. Nonetheless, this circumstance should not affect the results of the statistical analysis, given that those missed during the follow up period would not be different, in principle, from the groups of interest.

### Conclusions and implications

Our results suggest that SRH is a good indicator of mortality in persons who reside in care or nursing homes in the autonomous community of Madrid. Nevertheless, in the group of participants with the highest levels of disability and/or dementia, for whom there is a higher probability of not answering the item on the questionnaire, SRH might not be such a good predictor. This implies that, in populations with high prevalence of disability, associated or not with cognitive deterioration, the use of other markers for mortality would be more reliable than SRH.

## Data Availability

Data and material are available on reasonable request to the last author, Javier Damian (jdamian@isciii.es).

## Abbreviations

CHF: Congestive heart failure
CHP: Chronic health problems
CI: Confidence interval
COPD: Pulmonary obstructive disease
PR: Prevalence ratio
ref.: reference category
SD: Standard deviation
SRH: Self-rated health
